# Supplementary material for: Ethnic inequalities in palliative care prescribing in high income countries – a rapid systematic review
Source: BMC Palliat Care. 2025 Apr 26;24:119. doi: 10.1186/s12904-025-01742-1 (PMC12034175; doi:10.1186/s12904-025-01742-1)
Supplement: Supplementary file 1 — Supplementary Material 1. [file 12904_2025_1742_MOESM1_ESM.docx]

**Supplementary Tables**

**Supplementary Table 1: The Kunonga Framework for Extending Existing Approaches in Inequality/Inequity-Focused Evidence Syntheses**

| **Stage** | **Component** | **Description** |
| --- | --- | --- |
| Protocol Stage | Define Key Terms and Scope | In addition to standard scope-setting, clarify the primary focus on health inequality and/or health inequity, providing clear definitions and examples. This step ensures alignment with the review’s objectives and establishes priorities for eligibility criteria and subsequent analysis. |
|  | Frameworks and Theoretical Approach Selection | Select appropriate frameworks, such as PROGRESS Plus, incorporating both intersectional and life-course perspectives to address social determinants comprehensively. This integration enables an exploration of how health outcomes develop and vary across life stages and social contexts. |
|  | Developing a Logic Model | Develop a detailed logic model that visualises potential pathways through which intersecting social and structural factors, along with life-course elements, contribute to health disparities. This model may guide data collection, analysis, and interpretation, mapping cumulative impacts on health over time. |
| Data Extraction Stage | Mapping Data | Organise and categorise data by demographic, socioeconomic, and healthcare factors to reveal disparities in access and outcomes. |
| Analysis Stage | Intersectionality | Construct an intersectionality matrix to examine how overlapping factors such as race, gender, socioeconomic status, and age shape access to care and health outcomes. Applying structured subgroup analyses can further highlight barriers faced by specific groups and trends across these intersections. |
|  | Life-course Analysis | Adopt a life-course perspective by analysing health outcomes as they evolve across different life stages. Use frameworks such as the critical period model or accumulation model to assess how early-life exposures and cumulative experiences impact health trajectories over time, providing a holistic view of inequalities. |

**Supplementary Table 2: Overview of study results for medication prescribing and ethnicity**

| **Study ID** | **Risk of bias** | **N participants** | **Outcome measure** | **Analysis type** | **Outcome data** | | | | | |
| --- | --- | --- | --- | --- | --- | --- | --- | --- | --- | --- |
|  |  |  |  |  | **White/ White, non-Hispanic** | **Black** | **Hispanic/ Latino/ Latina** | **Asian/ Pacific Islander** | **Asian, other** | **Other** |
| **Antidepressants** | | | | | | | | | | |
| Allen et al., 2023 [31] | High | 74,309 | Use of pain medication - antidepressants | Adjusted analysis | Ref | Black/African American: OR 0.56 (95% CI 0.53 to 0.59; P < 0.05) | Hispanic: OR 0.77 (95% CI 0.73 to 0.82; P < 0.05) | Asian/Pacific Islander: 0.47 (95% CI 0.44 to 0.51; P < 0.05) | - | - |
| **Anorexia** | | | | | | | | | | |
| Saphire et al., 2020 [39] | High | 16,246 | Any anorexia medication receipt at EOL-1 | Adjusted analysis | Ref | Black, non Hispanic: aRR 1.49 (95 CI 1.32 to 1.69; P <0.001) | Hispanic: aRR 1.13 (95% CI 0.96 to 1.33; P 0.151) | - | Asian, other: aRR 1.90 (95% CI 1.16 to 2.19; P <0.001) | - |
| **Antipsychotics** | | | | | | | | | | |
| Allen et al., 2023 [31] | High | 74,309 | Use of pain medication - antipsychotics | Adjusted analysis | Ref | Black/African American: OR 0.54 (95% CI 0.52 to 0.57; P <0.05) | Hispanic: OR 0.74 (95% CI 0.70 to 0.78; P < 0.05) | Asian/Pacific Islander: OR 0.49 (95% CI 0.46 to 0.52; P < 0.05) | - | - |
|  |  |  | Use of pain medication - any psychotropic medication |  | Ref | Black/African American: OR 0.54 (95% CI 0.52 to 0.57; P <0.05) | Hispanic: OR 0.74 (95% CI 0.70 to 0.78; P < 0.05) | Asian/Pacific Islander: OR 0.49 (95% CI 0.46 to 0.52; P < 0.05) | - | - |
| **Anxiolytics** | | | | | | | | | | |
| Allen et al., 2023 [31] | High | 74,309 | Use of pain medication - anxiolytics | Adjusted analysis | Ref | Black/African American: OR 0.47 (95% CI 0.43 to 0.50; P < 0.05) | Hispanic: OR 0.66 (95% CI 0.62 to 0.71; P < 0.05) | Asian/Pacific Islander: OR 0.52 (95% CI 0.48 to 0.57; P < 0.05) | - | - |
| **Appetite stimulant** | | | | | | | | | | |
| Tabuyo-Martin et al., 2022 [40] | Fair | 186 | Appetite stimulant use - yes | Frequency data | White: 34 (24%) | Black: 19 (41%)  P = 0.038 | - | - | - | - |
| **Dyspnoea** | | | | | | | | | | |
| Saphire et al., 2020 [39] | High | 16,246 | Any dyspnoea medication receipt at EOL-1 | Adjusted analysis | Ref | Black, non Hispanic: aRR 0.80 (95% CI 0.70 to 0.92; P 0.002) | Hispanic: aRR 0.73 (95% CI 0.62 to 0.85; P <0.001) | - | Asian, other: aRR 0.73 (95% CI 0.63 to 0.85; P <0.001) | - |
| **Emotional distress** | | | | | | | | | | |
| Tabuyo-Martin et al., 2022 [40] | Fair | 186 | Agitation medication use - yes | Frequency data | White: 77 (55%) | Black: 14 (30%)  P = 0.088 | - | - | - | - |
| Saphire et al., 2020 [39] | High | 16,246 | Any emotional distress medication receipt at EOL-1 | Adjusted analysis | Ref | Black, non Hispanic: aRR 0.57 (95% CI 0.50 to 0.64; P <0.001) | Hispanic: aRR 0.62 (95% CI 0.53 to 0.72; P <0.001) | - | Asian, other: aRR 0.51 (95% CI 0.44 to 0.59; P <0.001) | - |
| **Nausea/vomiting** | | | | | | | | | | |
| Tabuyo-Martin et al., 2022 [40] | Fair | 186 | Antiemetic use - yes | Frequency data | White: 107 (76%) | Black: 35 (76%)  P = 1.00 | - | - | - | - |
| Saphire et al., 2020 [39] | High | 16,246 | Any nausea/vomiting medication receipt at EOL-1 | Adjusted analysis | Ref | Black, non Hispanic: aRR 0.76 (95% CI 0.64 to 0.89; P<0.001) | Hispanic: aRR 1.01 (95% CI 0.84 to 1.22; P<0.001) | - | Asian, other: aRR 0.75 (95% CI 0.62 to 0.90; P<0.001) | - |
| **Fatigue** | | | | | | | | | | |
| Saphire et al., 2020 [39] | High | 16,246 | Any fatigue medication receipt at EOL-1 | Adjusted analysis | Ref | Black/African American: aRR 1.48 (95% CI 1.28 to 1.72; P <0.001) | Hispanic: aRR 1.01 (95% CI 1.20 to 1.71; P <0.001) | - | Asian, other: aRR 1.38 (95% CI 1.16 to 1.63; P <0.001) | - |
| **Any pain medication (including opioids and non-opioids)** | | | | | | | | | | |
| Munir et al., 2023 [35] | Fair | 48,631 | Opioid use (1+ prescription) near EOL | Adjusted analysis | Ref | Black: OR 0.84 (95% CI 0.79 to 0.90; P <0.001) | Hispanic: OR 0.90 (95% CI 0.84 to 0.95; P <0.001) | - | Asian: OR 0.86 (95% CI 0.79 to 0.94; P <0.001) | Other: OR 0.83 (95% CI 0.74 to 0.93; P = 0.001) |
|  |  |  | Mean daily dose near EOL (percentage difference) |  | Ref | Black: -16.5% (95% CI -21.2 to -11.6%; P <0.001) | Hispanic: -19.1% (95% CI -23.5 to -14.6%; P <0.001) | - | Asian: -11.9% (95% CI -18.5 to -4.9%; P = 0.001) | Other: -7.8% (95% CI -16.4 to 1.5%; P = 0.099) |
| Rolnick et al., 2007 [37] | Fair | 421 | Likelihood of receiving high intensity pain medication 1-2 months before death | Unadjusted analysis | Ref | - | - | - | - | OR: 1.419 (95% CI 0.812 to 2.482; P = 0.219) |
| Tabuyo-Martin et al., 2022 [40] | Fair | 186 | Analgesic use – yes | Frequency data | White: 120 (86%) | Black: 37 (80%)  P = 0.482 | - | - |  |  |
| Allen et al., 2023 [31] | High | 74,309 | Use of pain medication – any pain medication | Adjusted analysis | Ref | Black/African American: OR 0.84 (95% CI 0.80 to 0.89; P < 0.05) | Hispanic: OR 1.07 (95% CI 1.04 to 1.17; P < 0.05) | Asian/Pacific Islander: OR 0.93 (95% CI 0.87 to 0.99; P < 0.05) | - | - |
|  |  |  | Use of pain medication - opioids |  | Ref | Black/African American: OR 0.84 (95% CI 0.79 to 0.88; P < 0.05) | Hispanic: OR 1.07 (95% CI 1.01 to 1.14; P < 0.05) | Asian/Pacific Islander: OR 0.84 (95% CI 0.79 to 0.90; P < 0.05) | - | - |
|  |  |  | Use of pain medication – non-opioids |  | Ref | Black/African American: OR 0.97 (95% CI 0.90 to 1.04; P NS) | Hispanic: OR 1.16 (95% CI 1.08 to 1.25; P<0.05) | 1.37 (95% CI 1.26 to 1.49; P<0.05) | - | - |
| Enzinger et al., 2023 [33] | High | 318,549 | Receipt of any opioid near EOL (absolute difference in percentage points) | Adjusted analysis (model 2) | Ref | Black: -5.4% (95% CI -6.0% to -4.8%) | Hispanic: -5.4% (95% CI -6.2% to -4.6%) | - | - | - |
|  |  |  | Receipt of any long-acting opioids near EOL (absolute difference in percentage points) |  | Ref | Black: -3.1% (95% CI -3.6 to -2.7%; P NR) | Hispanic: -2.4% (95% CI -3.0 to -1.9%; P NR) | - | - | - |
|  |  |  | Daily dose (morphine milligram equivalents) among opioid users near EOL |  | Ref | Black: -11.1 (95% CI -13.5 to -8.6; P NR) | Hispanic: -10.2 (95% CI -13.3 to -7.1; P NR) | - | - | - |
|  |  |  | Total dose (MMEs) filled by descendants near EOL |  | Ref | Black: -237 (95% CI -269 to -207; P NR) | Hispanic: -229 (95% CI -268 to -190; P NR) | - | - | - |
|  |  |  | Urine drug screen near EOL (absolute difference in percentage points) |  | Ref | Black: -0.2 (95% CI -0.5 to 0.0; P NR) | Hispanic: -1.1 (95% CI -1.5 to -0.8; P NR) | - | - | - |
| Sambamoorthi et al., 2000 [38] | High | 2131 | Use of pain medication in last 3 months of life | Adjusted analysis (model 3) | Ref | African-American: OR 0.63 (95% CI 0.48 to 0.83; P<0.05) | Latino/Latina: OR 0.79 (95% CI 0.56 to 1.11) | - | - | - |
| Saphire et al., 2020 [39] | High | 16,246 | Any pain medication at EOL-1 | Adjusted analysis | Ref | Black, non Hispanic: aRR 0.79 (95% CI 0.69 to 0.91; P 0.001) | Hispanic: aRR 0.74 (95% CI 0.63 to 0.87; P <0.001) | - | Asian, other: aRR 0.57 (95% CI 0.49 to 0.65; P <0.001) | - |
| Haider et al., 2017 [34] | High | 750 | Morphine equivalent daily dose | Frequency data | White: median 50 (IQR 20 to 100) | Black: median 40 (IQR 25 to 80) | Hispanic: median 40 (IQR 20 to 95) | - | - | Others: median 30 (IQR 15 to 70) |
| **Referral for palliative medicine** | | | | | | | | | | |
| Tabuyo-Martin et al., 2022 [40] | Fair | 186 | Palliative medicine referral | Frequency data | n=82  White: 57 (69.5%) | n=82  Black: 25 (30.5%)  P = 0.125 | - | - | - | - |
|  |  |  | No palliative medicine referral |  | n=104  White: 83 (79.8%) | n=104  Black: 21 (20.2%) |  |  |  |  |
| Abbreviations: aRR = adjusted risk ratio; CI = confidence interval; EOL = end of life; N = number; OR = odds ratio; ref = reference category | | | | | | | | | | |

**Supplementary Table 3: Overview of study results for medication prescribing and gender/sex**

| **Study ID** | **Risk of bias** | **N participants** | **Outcome measure** | **Analysis type** | **Outcome data** | |
| --- | --- | --- | --- | --- | --- | --- |
|  |  |  |  |  | **Female** | **Male** |
| **Anorexia** | | | | | | |
| Saphire et al., 2020 [39] | High | 16,246 | Any anorexia medication receipt at EOL-1 | Adjusted analysis | aRR 0.85 (95% CI 0.79 to 0.92; P<0.001) | Ref |
| **Dyspnoea** | | | | | | |
| Saphire et al., 2020 [39] | High | 16,246 | Any dyspnoea medication receipt at EOL-1 | Adjusted analysis | aRR 1.10 (95% CI 1.02 to 1.18; P = 0.019) | Ref |
| **Emotional distress** | | | | | | |
| Saphire et al., 2020 [39] | High | 16,246 | Any emotional distress medication receipt at EOL-1 | Adjusted analysis | aRR 1.35 (95% CI 1.27 to 1.45; P<0.001) | Ref |
| **Fatigue** | | | | | | |
| Saphire et al., 2020 [39] | High | 16,246 | Any fatigue medication receipt at EOL-1 | Adjusted analysis | aRR 0.89 (95% CI 0.82 to 0.97; P = 0.007) | Ref |
| **Nausea/vomiting** | | | | | | |
| Saphire et al., 2020 [39] | High | 16,246 | Any nausea medication receipt at EOL-1 | Adjusted analysis | aRR 1.27 (95% CI 1.17 to 1.39; P<0.001) | Ref |
| **Any pain medication** | | | | | | |
| Munir et al., 2023 [35] | Fair | 48,631 | Opioid use (1+ prescription) near EOL | Adjusted analysis | Ref | OR 0.92 (95% CI 0.89 to 0.96; P < 0.001) |
|  |  |  | Mean daily dose (MMED) near EOL (% difference) |  | Ref | 2.4% (-1.1 to 6.1%; P = 0.178) |
| Sambamoorthi et al., 2000 [38] | High | 2131 | Use of pain medication in last 3 months of life | Adjusted analysis (model 3) | OR 1.44 (95% CI 1.20 to 1.74; P<0.05) | Ref |
| Enzinger et al., 2023 [33] | High | 318,549 | Receipt of any opioid near EOL (absolute difference in percentage points) | Adjusted analysis (model 2) | Ref | 0.7% (95% CI 0.3% to 1.0%; P <0.001) |
|  |  |  | Receipt of any long-acting opioids near EOL |  | Ref | 1.1 (95% CI 0.9 to 1.4; P NR) |
|  |  |  | Daily dose (morphine milligram equivalents) among opioid users near EOL |  | Ref | 9.5 (95% CI 8.1 to 11.0; P NR) |
|  |  |  | Total dose (MMEs) filled by descendants near EOL |  | Ref | 140 (95% CI 122 to 158; P NR) |
|  |  |  | Urine drug screen near EOL |  | Ref | 0.7 (95% CI 0.6 to 0.9; P NR) |
| Saphire et al., 2020 [39] | High | 16,246 | Any pain medication at EOL-1 | Adjusted analysis | aRR 1.32 (95% CI 1.23 to 1.42; P<0.001) | Ref |
| Haider et al., 2017 [34] | High | 750 | Morphine equivalent daily dose | Frequency data | Median 45 (IQR 20 to 90)  P = 0.042 | Median 53 (IQR 23 to 100) |
| Abbreviations: aRR = adjusted risk ratio; CI = confidence interval; EOL = end of life; N = number; OR = odds ratio; ref = reference category | | | | | | |

**Supplementary Table 4: Overview of study results for medication prescribing and place of residence**

| **Study ID** | **Risk of bias** | **N participants** | **Outcome measure** | **Analysis type** | **Outcome data** | | | | | |
| --- | --- | --- | --- | --- | --- | --- | --- | --- | --- | --- |
|  |  |  |  |  | **Large metropolitan statistical area** | **Urban** | **Near urban** | **Rural** | **Less urban/ rural/ unknown**  **Other** | **Elsewhere** |
| **Anorexia** | | | | | | | | | |  |
| Saphire et al., 2020 [39] | High | 16,246 | Any anorexia medication receipt at EOL-1 | Adjusted analysis | Ref | aRR 0.89 (95% CI 0.76 to 1.04; P = 0.141) | - | - | aRR 1.14 (95% CI 1.02 to 1.27; P = 0.018) | - |
| **Dyspnoea** | | | | | | | | | | |
| Saphire et al., 2020 [39] | High | 16,246 | Any dyspnoea medication receipt at EOL-1 | Adjusted analysis | Ref | aRR 0.91 (95% CI 0.79 to 1.05; P = 0.196) | - | - | aRR 1.07 (95% CI 0.96 to 1.20; P = 0.212) | - |
| **Emotional distress** | | | | | | | | | | |
| Saphire et al., 2020 [39] | High | 16,246 | Any emotional distress medication receipt at EOL-1 | Adjusted analysis | Ref | aRR 0.96 (95% CI 0.84 to 1.09; P = 0.522) | - | - | aRR 1.02 (95% CI 0.92 to 1.13; P = 0.675) | - |
| **Nausea/vomiting** | | | | | | | | | | |
| Saphire et al., 2020 [39] | High | 16,246 | Any nausea/vomiting medication receipt at EOL-1 | Adjusted analysis | Ref | aRR 1.13 (95% CI 0.96 to 1.33; P = 0.134) | - | - | aRR 1.22 (95% CI 1.08 to 1.37; P = 0.001) | - |
| **Fatigue** | | | | | | | | | | |
| Saphire et al., 2020 [39] | High | 16,246 | Any fatigue medication receipt at EOL-1 | Adjusted analysis | Ref | aRR 0.80 (95% CI 0.67 to 0.95; P = 0.009) | - | - | aRR 0.91 (95% CI 0.80 to 1.03; P = 0.145) | - |
| **Any pain medication** | | | | | | | | | | |
| Munir et al., 2023 [35] | Fair | 48,631 | Opioid use (1+ prescription) near EOL | Adjusted analysis | - | Ref | - | OR 1.13 (95% CI 1.06 to 1.20; P <0.001) | - | - |
|  |  |  | Mean daily dose (MMED) near EOL (percentage difference) |  | - | Ref | - | 2.8% (95% CI -2.6 to 8.5%; P = 0.321) | - | - |
| Sambamoorthi et al., 2000 [38] | High | 2131 | Use of pain medication in last 3 months of life | Adjusted analysis (model 3) | - | - | OR 0.95 (95% CI 0.77 to 1.16) | - | - | Ref |
| Saphire et al., 2020 [39] | High | 16,246 | Any pain medication at EOL-1 | Adjusted analysis | Ref | aRR 1.16 (95% CI 1.01 to 1.35; P = 0.041) | - | - | aRR 1.22 (95% CI 1.09 to 1.36; P = 0.000) | - |
| Enzinger et al., 2023 [33] | High | 318,549 | Receipt of any opioid near EOL (absolute difference in percentage points) | Adjusted analysis | - | -3.9% (95% CI -4.3% to -3.5%; P NR) | - | Ref | - | - |
|  |  |  | Receipt of any long-acting opioids near EOL (absolute difference in percentage points) |  | - | -1.9% (95% CI -2.2 to -1.6%; P NR) | - | Ref | - | - |
|  |  |  | Daily dose (morphine milligram equivalents) among opioid users near EOL |  | - | -2.4% (95% CI -3.9 to -0.9%; P NR) | - | Ref | - | - |
|  |  |  | Total dose (MMEs) filled by descendants near EOL |  | - | -111 (95% CI -131 to -92; P NR) | - | Ref | - | - |
|  |  |  | Urine drug screen near EOL (absolute difference in percentage points) |  | - | -0.4% (95% CI -0.6 to -0.2%; P NR) | - | Ref | - | - |
| Abbreviations: aRR = adjusted risk ratio; CI = confidence interval; EOL = end of life; N = number; OR = odds ratio; ref = reference category | | | | | | | | | | |

**Supplementary Table 5: Overview of study results for medication prescribing and personal characteristics (age)**

| **Study ID** | **Risk of bias** | **N participants** | **Outcome measure** | **Analysis type** | **Outcome data** | | | | |
| --- | --- | --- | --- | --- | --- | --- | --- | --- | --- |
|  |  |  |  |  | **Age 66-69** | **Age 70-74** | **Are 75-79** | **80+** | **1 unit increase in age** |
| **Anorexia** | | | | | | | | | |
| Saphire et al., 2020 [39] | High | 16,246 | Any anorexia medication receipt at EOL-1 | Adjusted analysis | Ref | aRR 0.99 (95% CI 0.88 to 1.12; P = 0.901) | aRR 1.09 (95% CI 0.96 to 1.23; P = 0.187) | aRR 1.02 (95% CI 0.91 to 1.15; P = 0.698) | - |
| **Dyspnoea** | | | | | | | | | |
| Saphire et al., 2020 [39] | High | 16,246 | Any dyspnoea medication receipt at EOL-1 | Adjusted analysis | Ref | aRR 1.05 (95% CI 0.93 to 1.18; P = 0.443) | aRR 0.95 (95% CI 0.85 to 1.07; P = 0.424) | aRR 0.86 (95% CI 0.77 to 0.97; P = 0.011) | - |
| **Emotional distress** | | | | | | | | | |
| Saphire et al., 2020 [39] | High | 16,246 | Any emotional distress medication receipt at EOL-1 | Adjusted analysis | Ref | aRR 0.88 (95% CI 0.79 to 0.97; P = 0.012) | aRR 0.74 (95% CI 0.67 to 0.83; P <0.001) | aRR 0.68 (95% CI 0.61 to 0.75; P <0.001) | - |
| **Nausea/vomiting** | | | | | | | | | |
| Saphire et al., 2020 [39] | High | 16,246 | Any nausea/vomiting medication receipt at EOL-1 | Adjusted analysis | Ref | aRR 0.88 (95% CI 0.78 to 0.99; P 0.040) | aRR 0.78 (95% CI 0.68 to 0.88; P <0.001) | aRR 0.57 (95% CI 0.50 to 0.65; P <0.001) | - |
| **Fatigue** | | | | | | | | | |
| Saphire et al., 2020 [39] | High | 16,246 | Any fatigue medication receipt at EOL-1 | Adjusted analysis | Ref | aRR 0.92 (95% CI 0.82 to 1.04; P = 0.195) | aRR 0.78 (95% CI 0.69 to 0.89; P <0.001) | aRR 0.68 (95% CI 0.60 to 0.77; P < 0.001) | - |
| **Any pain medication** | | | | | | | | | |
| Munir et al., 2023 [35] | Fair | 48,631 | Opioid use (1+ prescription) near EOL | Adjusted analysis | - | - | - | - | OR 0.80 (95% CI 0.79 to 0.82; P < 0.001) |
|  |  |  | Mean daily dose (MMED) near EOL (% difference) |  | - | - | - | - | -20.1% (95% CI -21.1 to -19.1%; P < 0.001) |
| Saphire et al., 2020 [39] | High | 16,246 | Any pain medication at EOL-1 | Adjusted analysis | Ref | aRR 0.82 (95% CI 0.73 to 0.92; P = 0.001) | aRR 0.68 (95% CI0.60 to 0.76 ; P <0.001) | aRR 0.45 (95% CI 0.41 to 0.51; P < 0.001) | - |
| Abbreviations: aRR = adjusted risk ratio; CI = confidence interval; EOL = end of life; N = number; OR = odds ratio; ref = reference category | | | | | | | | | |

**Supplementary Table 6: Overview of study results for medication prescribing and personal characteristics (age at diagnosis)**

| **Study ID** | **Risk of bias** | **N participants** | **Outcome measure** | **Analysis type** | **Outcome data** | | | |
| --- | --- | --- | --- | --- | --- | --- | --- | --- |
|  |  |  |  |  | **Age 18-29** | **Age 30-39** | **Age 40-49** | **Age 50 and over** |
| **Any pain medication** | | | | | | | | |
| Sambamoorthi et al., 2000 [38] | High | 2131 | Use of pain medication in last 3 months of life – waiver/race interactions | Adjusted analysis | Ref | OR 1.08 (95% CI 0.83 to 1.40; P not statistically significant) | OR 1.14 (95% CI 0.85 to 1.51; P not statistically significant) | OR 0.96 (95% CI 0.63 to 1.44; P not statistically significant) |
| Abbreviations: CI = confidence interval; N = number; OR = odds ratio; ref = reference category | | | | | | | | |

**Supplementary Table 7: Overview of study results for medication prescribing and SES**

| **Study ID** | **Risk of bias** | **N participants** | **Outcome measure** | **Analysis type** | **Outcome data** |
| --- | --- | --- | --- | --- | --- |
| **Anorexia** | | | | | |
| Saphire et al., 2020 [39] | High | 16,246 | Any anorexia medication receipt at EOL-1 | Adjusted analysis | Poverty rates (census tract levels)  Ref - low census tract  5% to <10%: aRR 0.99 (95% CI 0.88 to 1.12; P 0.931)  10% to <20%: aRR 1.16 (95% CI 1.04 to 1.30; P = 0.010)  High (20% to 100%): aRR 1.32 (95% CI 1.17 to 1.49; P<0.001) |
| **Dyspnoea** | | | | | |
| Saphire et al., 2020 [39] | High | 16,246 | Any dyspnoea medication receipt at EOL-1 | Adjusted analysis | Poverty rates (census tract levels)  Ref - low census tract  5% to <10%: aRR 1.04 (95% CI 0.94 to 1.16; P = 0.439)  10% to <20%: aRR 1.03 (95% CI 0.92 to 1.14; P = 0.643)  High (20% to 100%): aRR 0.94 (95% CI 0.83 to 1.06; P = 0.322) |
| **Emotional distress** | | | | | |
| Saphire et al., 2020 [39] | High | 16,246 | Any emotional distress medication receipt at EOL-1 | Adjusted analysis | Poverty rates (census tract levels)  Ref - low census tract  5% to <10%: aRR 0.94 (95% CI 0.86 to 1.04; P = 0.228)  10% to <20%: aRR 0.86 (95% CI 0.78 to 0.95; P = 0.003)  High (20% to 100%): aRR 0.80 (95% CI 0.72 to 0.90l P <0.001) |
| **Nausea/vomiting** | | | | | |
| Saphire et al., 2020 [39] | High | 16,246 | Any nausea/vomiting medication receipt at EOL-1 | Adjusted analysis | Poverty rates (census tract levels)  Ref - low census tract  5% to <10%: aRR 0.98 (95% CI 0.87 to 1.11; P = 0.752)  10% to <20%: aRR 1.04 (95% CI 0.92 to 1.17; P = 0.547)  High (20% to 100%): aRR 0.99 (95% CI 0.87 to 1.14; P = 0.929) |
| **Fatigue** | | | | | |
| Saphire et al., 2020 [39] | High | 16,246 | Any fatigue medication receipt at EOL-1 | Adjusted analysis | Poverty rates (census tract levels)  Ref - low census tract  5% to <10%: aRR 0.92 (95% CI 0.81 to 1.03; P = 0.145)  10% to <20%: aRR 0.86 (95% CI 0.77 to 0.97; P = 0.015)  High (20% to 100%): aRR 0.89 (95% CI 0.78 to 1.01; P = 0.078) |
| **Any pain medication** | | | | | |
| Munir et al., 2023 [35] – poverty duration | Fair | 48,631 | Opioid use (1+ prescription) near EOL | Adjusted analysis | Poverty duration  Ref=never high poverty  Intermittent high poverty: 1.09 (95% CI 1.02 to 1.17; P = 0.014)  Persistent poverty: 1.18 (95% CI 1.09 to 1.27; P < 0.001) |
|  |  |  | Mean daily dose (MMED) near EOL - % difference |  | Poverty duration  Ref=never high poverty  Intermittent high poverty: -11.9% (95% CI -17.1 to -6.4%; P < 0.001)  Persistent poverty: -10.4% (95% CI -16.2 to -4.3%; P = 0.001) |
| Munir et al., 2023 [35] – dual eligibility for Medicare and Medicaid | Fair | 48,631 | Opioid use (1+ prescription) near EOL | Adjusted analysis | Dual eligibility for Medicare and Medicaid  Ref=non-dual  Dual: OR 1.15 (95% CI 1.10 to 1.21; P <0.001) |
|  |  |  | Mean daily dose (MMED) near EOL - % difference |  | Dual eligibility for Medicare and Medicaid  Ref=non-dual  Dual: 5.4% (95% CI 1.3 to 7.4%; P = 0.001) |
| Sambamoorthi et al., 2000 [38] | High | 2131 | Use of pain medication in last 3 months of life – waiver/race interactions | Adjusted analysis | African-American - ACCAP: 1.13 (95% CI 0.71 to 1.81; P not statistically significant)  Latino/Latina - ACCAP: 1.31 (95% CI 0.72 to 2.42; P not statistically significant) |
|  |  |  | Use of pain medication in last 3 months of life - waiver status |  | Waiver status  Ref=Non ACCAP  ACCAP: 2.30 (95% CI 1.59 to 3.36; P <0.05) |
| Saphire et al., 2020 [39] | High | 16,246 | Any pain medication at EOL-1 | Adjusted analysis | Poverty rates (census tract levels)  Ref - low census tract  5% to <10%: aRR 1.10 (95% CI 1.00 to 1.22; P = 0.051)  10% to <20%: aRR 1.10 (95% CI 1.00 to 1.21; P = 0.055)  High (20% to 100%): aRR 1.21 (95% CI 1.08 to 1.35; P = 0.001) |
| Enzinger et al., 2023 [33] – low income (dually eligible for Medicare and Medicaid) | High | 318,549 | Receipt of any opioid near EOL (absolute difference in percentage points) | Adjusted analysis | Low income (dually eligible for Medicare and Medicaid)  Ref = non-dual eligible  Dual eligible: 6.1% (95% CI 5.7% to 6.5%; P NR) |
|  |  |  | Receipt of long acting opioids near EOL |  | Low income (dually eligible for Medicare and Medicaid)  Ref = non-dual eligible  Dual eligible: 2.1 (95% CI 1.7 to 2.4; P NR) |
|  |  |  | Daily dose (morphine milligram equivalents) among opioid users near EOL |  | Low income (dually eligible for Medicare and Medicaid)  Ref = non-dual eligible  Dual eligible: 5.8 (95% CI 4.3 to 7.4; P NR) |
|  |  |  | Total dose (morphine milligram equivalents) filled by descendants near EOL |  | Low income (dually eligible for Medicare and Medicaid)  Ref = non-dual eligible  Dual eligible: 192 (95% CI 171 to 213; P NR) |
| Enzinger et al., 2023 [33] - community-level deprivation (SDI quintile) | High | 318,549 | Receipt of any opioid near EOL (absolute difference in percentage points) | Adjusted analysis | Community-level deprivation (SDI quintile)  Ref = Q1 lowest)  Q2: -0.3 (95% CI -0.8 to 0.2; P NR)  Q3: 0.0 (95% CI -0.5 to 0.6; P NR)  Q4: -0.6 (95% CI -1.1 to 0.0; P NR)  Q5 (highest): -1.3 (95% CI -1.9 to -0.8; P NR) |
|  |  |  | Receipt of long acting opioids near EOL |  | Community-level deprivation (SDI quintile)  Ref = Q1 lowest  Q2: 0.0 (95% CI -0.4 to 0.3; P NR)  Q3: 0.0 (95% CI -0.5 to 0.3; P NR)  Q4: -0.8 (95% CI -1.1 to -0.4; P NR)  Q5 (highest): -1.4 (95% CI -1.8 to -1.0; P NR) |
|  |  |  | Daily dose (morphine milligram equivalents) among opioid users near EOL |  | Community-level deprivation (SDI quintile)  Ref = Q1 lowest  Q2: 0.6 (95% CI -1.4 to 2.6; P NR)  Q3: -0.3 (95% CI -2.3 to 1.7; P NR)  Q4: -2.6 (95% CI -4.7 to -0.5; P NR)  Q5 (highest): -3.0 (95% CI -5.2 to -0.8; P NR) |
|  |  |  | Total dose (morphine milligram equivalents) filled by descendants near EOL |  | Community-level deprivation (SDI quintile)  Ref = Q1 lowest  Q2: -0.6 (95% CI -26 to 25; P NR)  Q3: -4.2 (95% CI -30 to 22; P NR)  Q4: -41 (95% CI -68 to -14; P NR)  Q5 (highest): -64 (95% CI -92 to -35; P NR) |
|  |  |  | Urine drug screen near EOL |  | Community-level deprivation (SDI quintile)  Ref = Q1 lowest  Q2: 0.1 (95% CI 0.1 to 0.3; P NR)  Q3: 0.2 (95% CI 0.0 to 0.4; P NR)  Q4: 0.4 (95% CI 0.1 to 0.6; P NR)  Q5 (highest): 0.6 (95% CI 0.4 to 0.9; P NR) |
| Abbreviations: ACCAP: HIV/AIDS specific Medicaid Home- and Community-Based Waiver Program; aRR = adjusted risk ratio; CI = confidence interval; EOL = end of life; N = number; OR = odds ratio; ref = reference category | | | | | |

**Supplementary Table 8: Overview of study results for medication prescribing and social capital**

| **Study ID** | **Risk of bias** | **N participants** | **Outcome measure** | **Analysis type** | **Outcome data** | |
| --- | --- | --- | --- | --- | --- | --- |
|  |  |  |  |  | **Married** | **Unmarried** |
| **Anorexia** | | | | | | |
| Saphire et al., 2020 [39] | High | 16,246 | Any anorexia medication receipt at EOL-1 | Adjusted analysis | Ref | aRR 0.84 (95% CI 0.78 to 0.91; P <0.001) |
| **Dyspnoea** | | | | | | |
| Saphire et al., 2020 [39] | High | 16,246 | Any dyspnoea medication receipt at EOL-1 | Adjusted analysis | Ref | aRR 0.92 (95% CI 0.85 to 1.00; P = 0.051) |
| **Emotional distress** | | | | | | |
| Saphire et al., 2020 [39] | High | 16,246 | Any emotional distress medication receipt at EOL-1 | Adjusted analysis | Ref | aRR 0.92 (95% CI 0.86 to 0.99; P = 0.026) |
| **Nausea/vomiting** | | | | | | |
| Saphire et al., 2020 [39] | High | 16,246 | Any nausea/vomiting medication receipt at EOL-1 | Adjusted analysis | Ref | aRR 0.90 (95% CI 0.83 to 0.98; P = 0.021) |
| **Fatigue** | | | | | | |
| Saphire et al., 2020 [39] | High | 16,246 | Any fatigue medication receipt at EOL-1 | Adjusted analysis | Ref | aRR 0.96 (95% CI 0.88 to 1.05; P = 0.376) |
| **Any pain medication** | | | | | | |
| Saphire et al., 2020 [39] | High | 16,246 | Any pain medication at EOL-1 | Adjusted analysis | Ref | aRR 0.98 (95% CI 0.91 to 1.05; P = 0.558) |
| Abbreviations: aRR = adjusted risk ratio; CI = confidence interval; EOL = end of life; N = number; ref = reference category | | | | | | |
